# Estimating disparities in breast cancer screening programs towards mortality, case fatality, and DALYs across BRICS-plus

**DOI:** 10.1186/s12916-023-03004-4

**Published:** 2023-09-01

**Authors:** Sumaira Mubarik, Saima Shakil Malik, Zhang Yanran, Eelko Hak, Nawsherwan, Fang Wang, Chuanhua Yu

**Affiliations:** 1 Department of Epidemiology and Biostatistics, School of Public Health, Wuhan University, 185 Donghu Road, Wuhan, 430071 Hubei China; 2 PharmacoTherapy, -Epidemiology and -Economics, Groningen Research Institute of Pharmacy, University of Groningen, Groningen, The Netherlands; 3 Center for Biotechnology & Genomic Medicine (CBGM) Medical College of Georgia Augusta University, 1462 Laney Walker Blvd, Augusta, GA 30912-4810 USA; 4 Wuhan Jinyintan Hospital, Tongji Medical College of Huazhong University of Science and Technology; Hubei Clinical Research Center for Infectious Diseases; Wuhan Research Center for Communicable Disease Diagnosis and Treatment, Chinese Academy of Medical Sciences; Joint Laboratory of Infectious Diseases and Health, Wuhan Institute of Virology and Wuhan Jinyintan Hospital, Chinese Academy of Sciences, Wuhan, Hubei China; 5 Xiamen Cardiovascular Hospital of Xiamen University, School of Medicine, Xiamen University, Xiamen, Fujian 361000 China; 6 Department of Biostatistics, School of Public Health, Xuzhou Medical University, Xuzhou, 221004 Jiangsu China

**Keywords:** Breast cancer screening, Mortality, Case fatality, Disability, CVD, BRICS-plus

## Abstract

**Background:**

Numerous studies over the past four decades have revealed that breast cancer screening (BCS) significantly reduces breast cancer (BC) mortality. However, in BRICS-plus countries, the association between BCS and BC case fatality and disability are unknown. This study examines the association of different BCS approaches with age-standardized mortality, case-fatality, and disability-adjusted life years (DALYs) rates, as well as with other biological and sociodemographic risk variables, across BRICS-plus from a national and economic perspective.

**Methods:**

In this ecological study applying mixed-effect multilevel regression models, a country-specific dataset was analyzed by combining data from the Global Burden of Disease study 2019 on female age-standardized BC mortality, incidence, and DALYs rates with information on national/regional BCS availability (against no such program or only a pilot program) and BCS type (only self-breast examination (SBE) and/or clinical breast examination (CBE) [SBE/CBE] versus SBE/CBE with mammographic screening availability [MM and/or SBE/CBE] versus SBE/CBE/mammographic with digital mammography and/or ultrasound (US) [DMM/US and/or previous tests] in BRICS-plus countries.

**Results:**

Compared to self/clinical breast examinations (SBE/CBE) across BRICS-plus, more complex BCS program availability was the most significant predictor of decreased mortality [MM and/or SBE/CBE: − 2.64, *p* < 0.001; DMM/US and/or previous tests: − 1.40, *p* < 0.001]. In the BRICS-plus, CVD presence, high BMI, second-hand smoke, and active smoking all contributed to an increase in BC mortality and DALY rate. High-income and middle-income regions in BRICS-plus had significantly lower age-standardized BC mortality, case-fatality, and DALYs rates than low-income regions when nationwide BC screening programs were implemented.

**Conclusions:**

The availability of mammography (digital or traditional) and BCS is associated with breast cancer burden in BRICS-plus countries, with regional variations. In light of high-quality evidence from previous causal studies, these findings further support the preventive role of mammography screening for BCS at the national level. Intervening on BCS related risk factors may further reduce the disease burden associated with BC.

**Supplementary Information:**

The online version contains supplementary material available at 10.1186/s12916-023-03004-4.

## Background

Breast cancer (BC) is the primary cause of cancer related mortality and morbidity worldwide among women, accounting for 1/8 of all cancer diagnoses in 2020, with 2.3 million new cases, and it is among the top causes of mortality in low- and low-middle-income countries [1]. Breast cancer is the leading cause of cancer death among women in developing countries with worse disease outcomes and being the second most common cause of deaths from cancer in the developed countries [1].

Breast cancer screening is an effective, simple, and cost-effective method of screening asymptomatic women for early detection, diagnosis, and treatment; the goal is to reduce breast cancer mortality in the population [2]. This is true despite the fact that population-based mammography has been used extensively in high-income countries for more than 30 years and that these countries have more resources in terms of qualified doctors and mammogram units per capita, which increases the likelihood of finding breast cancer through increased screening [3, 4]. However, there is limited evidence indicating whether or not it is cost-effective in low middle-income countries [5].

Many imaging experts from the Society of Breast Imaging (SBI) and American Cancer Research (ACR) have examined extensive literature on ultrasound, digital mammography, and magnetic resonance imaging to elucidate their importance in early-stage diagnosis. Mammography is the principal diagnostic tool for early detection of breast cancer with average risk which plays potent role in diagnosing smaller size tumors with less nodal metastasis and lower grade tumor progression, leading to effective treatment modalities. Mammographic screening offers decrease in advanced stage/metastatic disease directly linked to considerably declined BC mortality. Both SBI and ACR recommended annual BC screening at the age of 40 years to get maximum benefits and lessen the disease severity. But it is advised to consider both risks and benefits before assisting women in getting informed choices [2, 6, 7].

Annual mammographic screening before the diagnosis of BC serves as potent increased survival predictor and women who had missed one or more of their last 5 annual screening mammograms had 2.3-fold increased mortality rates [8]. Contradictory results have been reported regarding the link between breast cancer screening and reduced chance of breast cancer related death [9]. One of the meta-analyses conducted in 2016 has concluded no association of mortality and screening programs for women aged 39 to 49 years [10], whereas women in the age range of 40–74 years with BC screening every year or two were reported to have 40% reduced chances of death from breast cancer making screening a good choice towards better health [11]. In addition, some scholars disagree with the use of BC mortality alone to evaluate the benefits and harms of screening and believe that the increase in other mortality caused by overdiagnosis, and overtreatment caused by BCS should be objectively analyzed [12].

Clinicians are encouraging to explore and develop early diagnostic approaches for precise diagnosis of early-stage BC and increasing the access of common people to basic health necessities and diagnostic services so that timely treatment can be provided. Improvement of patient’s survival is the major therapeutic aim of oncologists preferably depending on early-stage diagnosis [13].

Both oncologists and researchers have agreed on the effectiveness and success of breast cancer screening (BCS) from the last four decades. One of the recent studies supported the importance of BCS with remarkable decrease in BC mortality rates in the countries having different kinds of screening programs available. A worldwide review of BCS studies has confirmed substantially decreased BC mortality in routine health care settings [14].

Brazil, Russia, India, China, and South Africa (BRICS) constitute an economic and political grouping of countries enduring rapid economic progress keeping nearly half of the global population [15]. BRICS has uniqueness of having leading economic countries in its region or sub-region. Taking BRICS to a step further, BRICS-plus concept is recently introduced to establish a new economical platform for creating bilateral and regional alliances across continents aiming to promote growth in all fundamentals of life [16]. In 2019, about 1.98 million women worldwide were diagnosed with breast cancer and 690,000 died, of which BRICS countries accounted for 45% of new cases and 51% of deaths [12]. In 2012 alone, BC-related loss of female productivity in the BRICS countries reached 2.1 billion, ranking first among female cancers [17].

Although the BRICS countries cooperate in the field of public health and strive to achieve health equity, they still face major public health challenges due to late diagnosis and unavailability of proper resources. Addressing the challenge of increasing BC burden requires a multifaceted approach, including prevention, early diagnosis, better care, and modern treatment options for patients. At the same time, disease burden and medical resources vary across countries, and it is unclear which BCS programs are most effective and safe either mammography or magnetic resonance imaging (MRI) [18]. Although mammography serves as a baseline diagnosis of BC, it still comes up with certain risks such as overdiagnosis, overtreatment, and false-positive results leading to follow-ups and transient anxiety as well [19]. Breast MRI and needle biopsy are the choices for accurate diagnosis mostly for the women who have a high risk of BC development and monitoring treatment response in patients undergoing neoadjuvant chemotherapy. Although MRI have greater sensitivity and accuracy than mammography, it is still reported to have false-positive results putting extra pressure on patient’s health [20].

No comprehensive and systematic studies are available in BRICS-plus countries regarding the impact of BC screening on mortality, case fatality rate, and disability-adjusted life-years (DALYs). The burden of breast cancer among women in BRICS-plus countries is high, and medical resources are extremely imbalanced. Therefore, the aim of this ecological study was to examine the associations between BCS methods and age-standardized mortality, case fatality rate, DALYs, and other relevant sociodemographic and biological risk variables in BRICS-plus countries and economies.

## Methods

### Data sources and estimation of study variables

In this study, we utilized the Global Burden of Disease (GBD) 2019 estimates [21], which encompass age-standardized rates (ASR) for female BC mortality, incidence, and DALYs, to examine the chronological patterns of risk factors and comorbidities associated with BC across the BRICS-plus nations in relation to their socioeconomic status [22]. The GBD 2019 articles explain how incidence rates, mortality rates, years of life Lost (YLLs), years living with disability (YLDs), and DALYs are calculated as well as other analytical methods to compare morbidity and mortality from specific diseases and injuries [22, 23]. The International Classification of Disease (ICD) coding system (ICD-9 and ICD-10 codes) [24] is specifically used in the GBD to describe death due to BC, and standard modeling procedures are used to estimate BC-specific mortality [22, 24]. The GBD study computed DALYs, a health metric, for each age, sex, and state cause stratum by combining fatal (YLL) and nonfatal (YLD) components [23]. The GBD study uses epidemiological data from thorough literature studies, health surveys, and other sources to estimate cause-specific and sequela-specific prevalence and incidence. The study used Bayesian meta-regression compartmental modeling in DisMod-MR 2.1 the most [25]. Additionally, as described in earlier papers, the GBD study devised and implemented disability weights for each distinct health condition [26, 27]. Using a microsimulation framework, the study multiplied the prevalence and accompanying disability weights for each cause’s sequelae to calculate YLDs [23]. The aforementioned methodology was applied to the current investigation to extract the causes of YLDs and DALYs, including neoplasms, diabetes, kidney disease, and cardiovascular illnesses.

### Socioeconomic indicators and estimate of risk variables

The World Bank ranked (high, moderate, or low) each nation's income in 2018–2019 [28]. The GBD 2017 comparative risk assessment divided risk variables and clusters into behavioral, environmental/occupational, and metabolic categories. Methods akin to those employed for nonfatal models were used to assess and model data on risk factor exposure levels, focusing on accurately fitting exposure distributions among continuous and polytomous risk factors. Standard GBD comparative risk assessment methods were used to calculate quantitative relative risks for each risk outcome pair, and population attributable fraction statistics were derived [29].

The severity of a risk's contribution to disease burden and the degree of exposure per risk level were used to calculate summary exposure values (SEVs) for risk factors. The SEV score ranges from 0 to 1, with “0” indicating no excess risk for a population and “1” indicating the highest risk. SEV is presented as a percentage, with 0% being the lowest value and 100% being the highest. Based on the latest GBD 2017 methodology, this study risk factor analysis focused on risk factors for BC [30–34], such as high body mass index (BMI), low physical activity, smoking habits, and second-hand smoke exposure.

For the current analysis, data on BC outcomes were gathered for 35 BRICS-plus nations as unit of analysis from 1990 to 2019 based on yearly death, incidence, and DALYs age standardized rate (ASR, per 100 k person-years). For instance, the Mortality Information System of the Ministry of Health in Brazil provided the majority of the original data [35]; the Center for Demographic Research at the New Economic School in Russia provided the mortality by region, age, sex, and cause of death reports [36]; the Indian Sample Registration System and Registrar General of India [37]; and the Disease Surveillance Points, Maternal and Child Surveillance System, Chinese Center for Disease Control a Estimates from GBD relied on advanced statistical modeling to address scant and frequently inconsistent data because there were lacking data on numerous diseases, injuries, and risk variables from numerous nations [38]. The sociodemographic index (SDI) divides countries into five quintiles based on national per capita income, average years of schooling for those over 15, and total fertility rate. 0 to 1 indicates the least to most developed.

### Breast cancer screening (BCS) information

Information regarding BCS, such as the types of screening methods employed (e.g., solely self-breast examination (SBE) and/or clinical breast examination (CBE) [SBE/CBE] versus SBE/CBE with mammographic screening availability [MM and/or SBE/CBE] versus SBE/CBE/mammographic with digital mammography and/or ultrasound (US) breast screening availability [DMM/US and/or previous tests], as well as the presence or absence of BCS programs (or whether programs are only pilot or opportunistic BCS initiatives), was collected and verified from multiple sources. These sources included the World Health Organization (WHO) Global Health Observatory [39], the WHO cancer country profiles (https://www.who.int/cancer/country-profiles/en/), OECD Health Statistics 2020 data on BCS (http://www.oecd.org/health/health-data.htm), International Agency for Research on Cancer/WHO IARC Handbooks of Cancer Prevention [40, 41], related literature [42–46], and internet searches on BCS for each country. In cases where data were incomplete or unavailable, WHO Collaborating Centers in a country were consulted for clarification. One hundred thirty of the 194 nations for which data were gathered had complete information. Out of 130, we choose 35 BRICS-plus nations for the extraction of the BCS data. Additional file 1: Table. SI has the entire list.

### Preliminary investigation

Descriptive statistics (boxplots) were used to depict trends in age-standardized BC mortality, incidence, and case fatality rates among BRICS-plus countries. Divide the age-standardized death rate by the incidence rate and multiply by 100 to determine the case-fatality percentage (CFP) [47]. The relationship between SDI and BC outcomes in relation to various BC screening methods was assessed using the Spearman correlation coefficient (*r*) between 1990 and 2019.

### Linear mixed effect regression analysis

Mixed-effect multilevel regression models were used to examine the relationship between female age-adjusted BC mortality, case fatality, and DALYs rates (as outcomes) and the presence of BCS screening programs at regional and national levels, as well as the specific BCS tests used in each country, after accounting for various risk factors (e.g., smoking habits, low physical activity, and others) [40, 48]. A second round of analysis was done in connection to national income (by income-levels/groups). Country-year data were handled as the first level of analysis in the multilevel analysis, while repeated measures of nations as aggregated data were treated as the second level of analysis. Maximum likelihood estimation was applied in this context. Using the R package lme4, linear mixed-model analyses were carried out. This examination of secondary, publicly available data did not require ethical approval or participant agreement.

## Results

### BC mortality and DALYs and its attributable risk factors across BRICS-plus

Various factors have been statistically examined as potent BC mortality predictors and among them breast screening is considered as the most clinically significant, where mammograph availability (MM and/or SBE/CBE) other than SBE/CBE (as opposed to only SBE or CBE examination) was associated with lower mortality rate (− 2.64, *p* < 0.001). High body mass index, smoking including second-hand smoke, diabetes, and cardiovascular diseases were also found to be associated with increased BC mortality. Table 1 shows the descriptive of predictor variables and their relationship with age-standardized BC mortality. Digital mammography (DMM/US and/or previous tests), as the most commonly used diagnostic/screening test, reduced the age-standardized mortality (− 1.40, *p* < 0.001) of BC compared to only SBE/CBE. Moreover, the availability of national and regional screening programs, as opposed to no or pilot/opportunistic programs, was significantly related to decreased BC mortality rates (national − 1.52, *p* < 0.001; regional − 1.40, *p* < 0.001, Table 1).

**Table 1:** Age-standardized female breast cancer rates in relation to biological, metabolic, and sociodemographic risk factors and breast cancer screening programs across BRICS-plus

	**Model 1 (breast cancer mortality)**			**Model 2 (breast cancer DALYs)**		
**Predictors**	**Coefficients**	***t*-value**	***p*-value**	**Coefficients**	***t*-value**	***p*-value**
Cardiovascular diseases	0.04	12.32	*p* < 0.001	0.01	1.42	0.64
Diabetes and kidney diseases	0.02	10.54	*p* < 0.001	0.09	24.65	*p* < 0.001
Neoplasms	0.06	18.40	*p* < 0.001	0.10	31.45	*p* < 0.001
High body-mass index	0.90	40.14	*p* < 0.001	17.58	26.87	*p* < 0.001
Low physical activity	− 0.02	− 0.71	0.48	− 0.28	− 0.42	*p* < 0.001
Secondhand smoke	0.40	18.03	*p* < 0.001	14.35	21.93	*p* < 0.001
Smoking	0.32	14.48	*p* < 0.001	9.73	14.86	*p* < 0.001
SBE/CBE	Reference			Reference		
MM and/or SBE/CBE	− 2.64	− 39.09	*p* < 0.001	− 16.66	− 37.99	*p* < 0.001
DMM/US and/or previous tests	− 1.40	− 18.77	*p* < 0.001	− 10.35	− 18.10	*p* < 0.001
No country/pilot screening program^a^	Reference			Reference		
National -wise screening program	− 1.52	− 22.93	*p* < 0.001	− 12.98	− 21.29	*p* < 0.001
Regional-wise screening program	− 1.40	− 18.77	*p* < 0.001	− 10.35	− 18.10	*p* < 0.001

Cardiovascular diseases, diabetes and kidney disease, and neoplasms are expressed as age-standardized years lived with disability. Neoplasms estimates exclude breast cancer. High body-mass index, low physical activity, secondhand smoke, and smoking are expressed as age standardized summary exposure values (SEVs; range 0–100)

*CBE* clinical breast examination, *DMM* digital mammography, *DMM/US* digital mammography and/or ultrasound, *LDL-c* low-density lipoprotein-cholesterol, *MM* mammography, *SBE* self-breast examination, *SBE/CBE tests* self-breast and/or clinical breast examination, *SEV* summary exposure value; *US*, ultrasound

^a^No country program or existence of an opportunistic or pilot screening program

Aforementioned variables exhibited similar results in relation to age-standardized BC DALYs as they did with mortality, but with different magnitudes. The availability of BCS exams with mammography (MM and/or SBE/CBE) and digital mammography (DMM/US and/or previous tests) decreased DALYs by − 16.66 and − 10.35 (for both, *p* < 0.001), respectively, compared to only SBE/CBE tests. The presence of national or regional BCS programs was also connected to age-standardized BC disability.

Furthermore, factors positively predicting age-standardized BC DALYs included actual smoking habits, secondhand smoke exposure, high body mass index, diabetes, and kidney diseases. In contrast, low physical activity was inversely associated with DALYs (− 0.28, *p* < 0.001) (Table 1).

### BC trends in relation to BC screening program by BRICS-plus countries

**Fig. 1:**
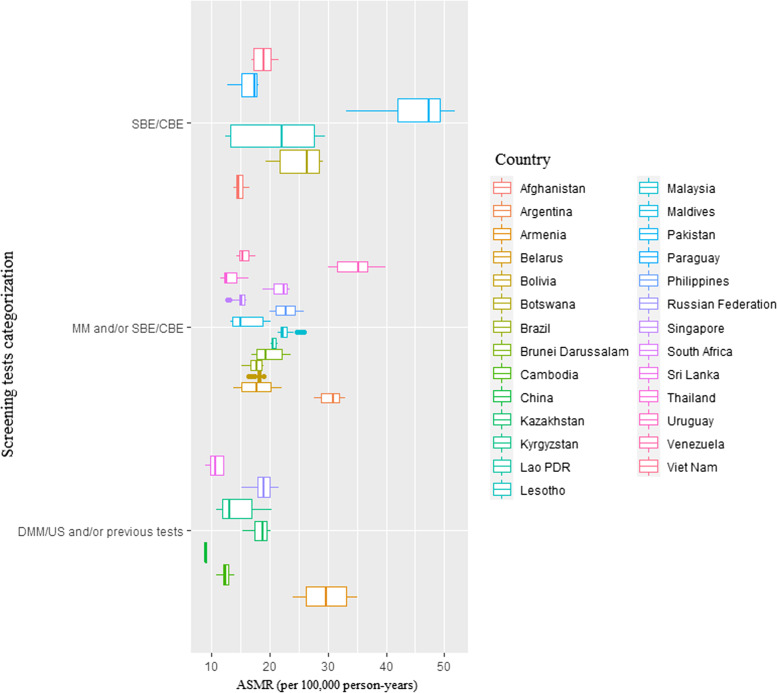
Age-standardized mortality rate (ASMR) 1990–2019 by type of screening test among BRICS-plus countries. CBE, clinical breast examination; DMM, digital mammography; DMM/US, digital mammography and/or ultrasound; MM, mammography; SBE, self-breast examination; SBE/CBE tests, self-breast examination and/or clinical breast examination; US, ultrasound

**Fig. 2:**
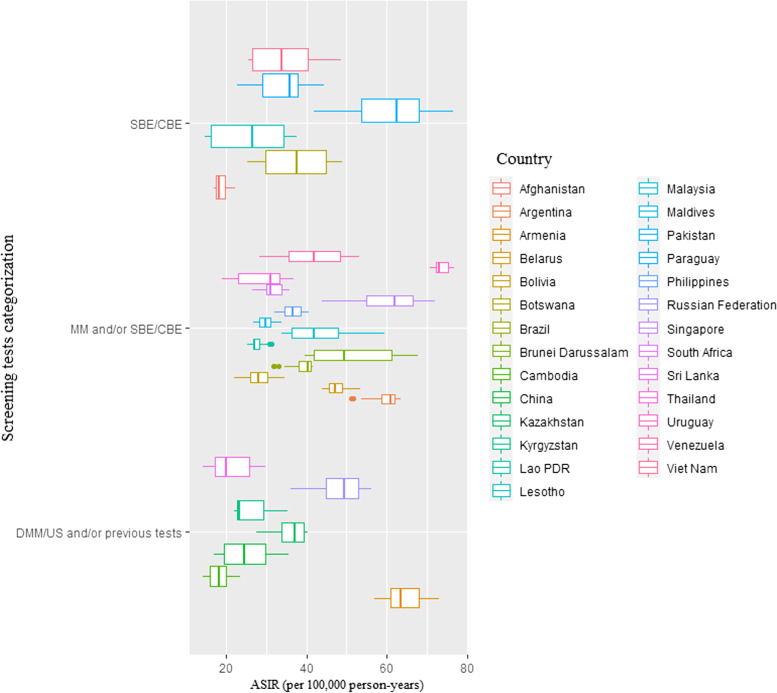
Age-standardized incidence rate (ASIR) 1990–2019 by type of screening test among BRICS-plus countries. CBE, clinical breast examination; DMM, digital mammography; DMM/US, digital mammography and/or ultrasound; MM, mammography; SBE, self-breast examination; SBE/CBE tests, self-breast examination and/or clinical breast examination; US, ultrasound

**Fig. 3:**
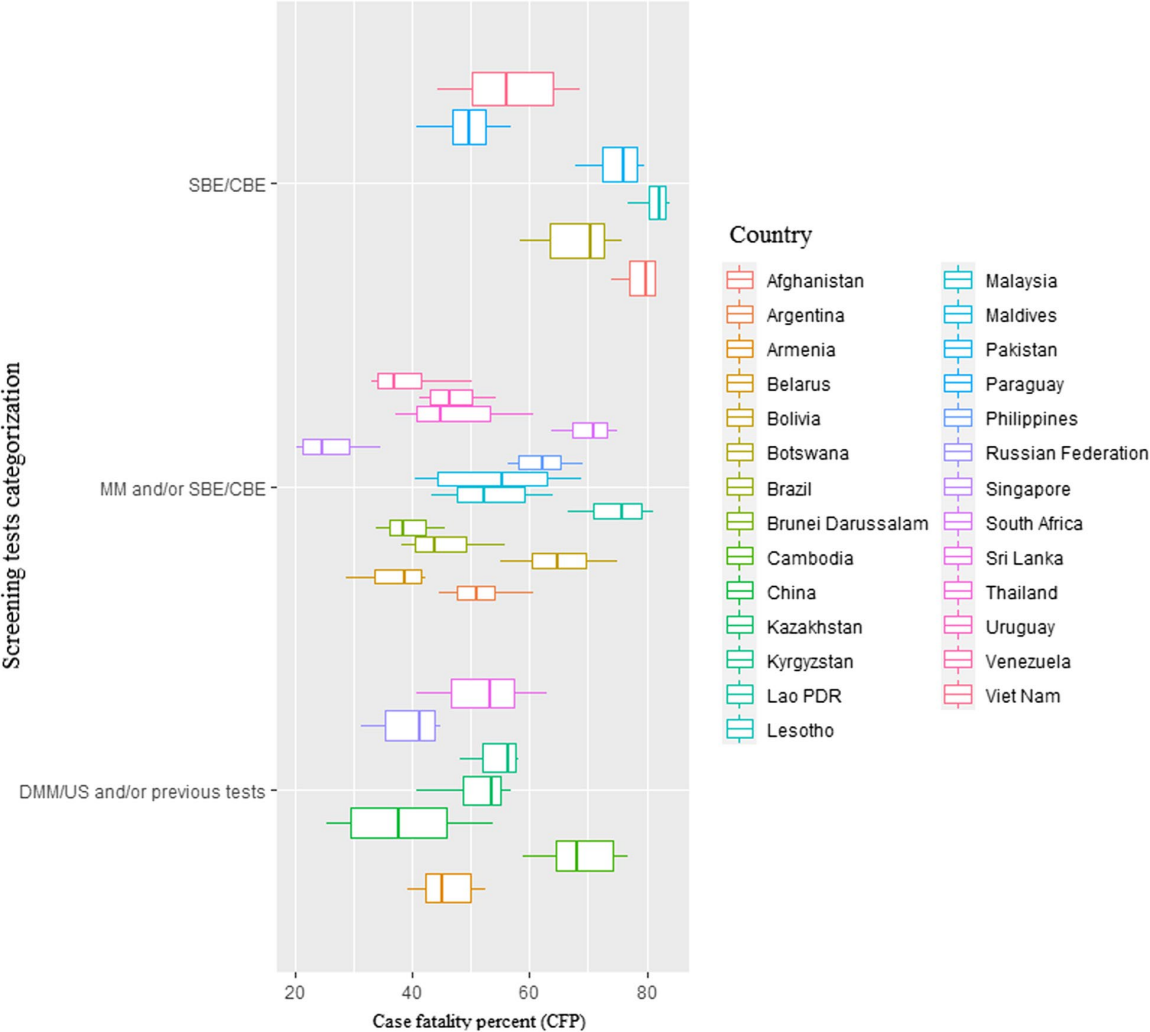
Case fatality percent (CFP) 1990–2019 by type of screening test among BRICS-plus countries. CFP indicate age-standardized mortality to incidence ratio and multiply by 100. CBE, clinical breast examination; DMM, digital mammography; DMM/US, digital mammography and/or ultrasound; MM, mammography; SBE, self-breast examination; SBE/CBE tests, self-breast examination and/or clinical breast examination; US, ultrasound

**Fig. 4:**
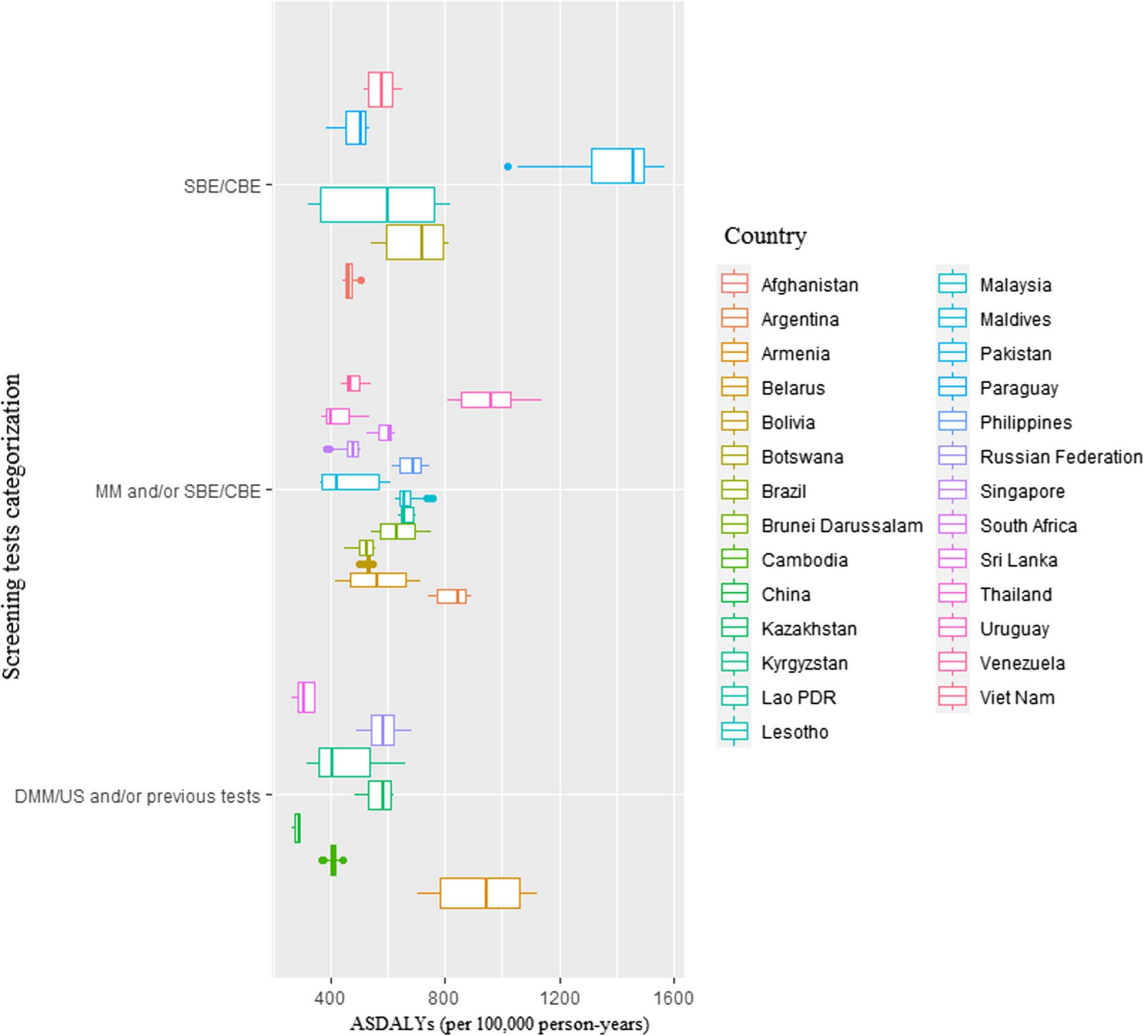
Age-standardized disability adjusted life years (ASDALYs) rate 1990–2019 by type of screening test among BRICS-plus countries. CBE, clinical breast examination; DMM, digital mammography; DMM/US, digital mammography and/or ultrasound; MM, mammography; SBE, self- breast examination; SBE/CBE tests, self-breast examination and/or clinical breast examination; US, ultrasound

The association between BC screening (comparing SBE/CBE to MM and/or SBE/CBE to DMM/US and/or previous tests) and mortality, incidence, case fatality percentage (CFP), and disability-adjusted life years (DALYs) was also shown by country from 1990 to 2019, highlighting variations in BC outcomes across different BRICS-plus countries (Figs. 1, 2, 3, and 4).

**Fig. 5:**
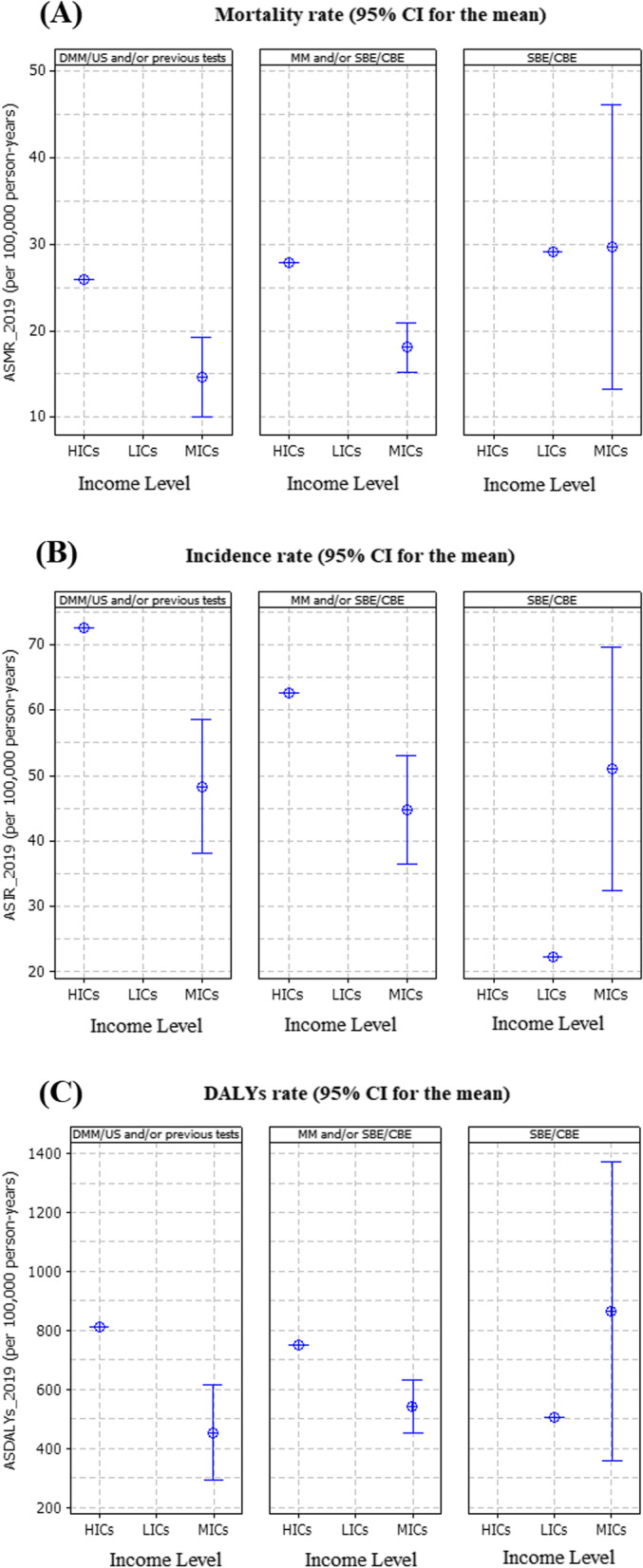
Age-standardized **A** mortality rate, **B** incidence rate, **C** disability-adjusted life years (DALYs) in year 2019 by type of screening test and income level/group among BRICS-plus countries. CBE, clinical breast examination; DMM, digital mammography; DMM/US, digital mammography and/or ultrasound; MM, mammography; SBE, self-breast examination; SBE/CBE tests, self-breast examination and/or clinical breast examination; US, ultrasound

The BRICS-plus nations in 2019 that used SBE/CBE/mammographic with digital mammography and/or ultrasound (US) [DMM/US and/or previous tests] were most of middle-income countries (MICs) as well as high-income countries (HICs) and had lower age-standardized mortality rate (ASMR) and age-standardized disability-adjusted life year (ASDALYs) than other screening programs. In contrast to SBE/CBE, the high-income countries (HICs) and MICs in BRICS-plus with access to MM and/or SBE/CBE and DMM/US and/or previous tests had greater age-standardized incidence rate (ASIR) in 2019 (Fig. 5).

**Fig. 6:**
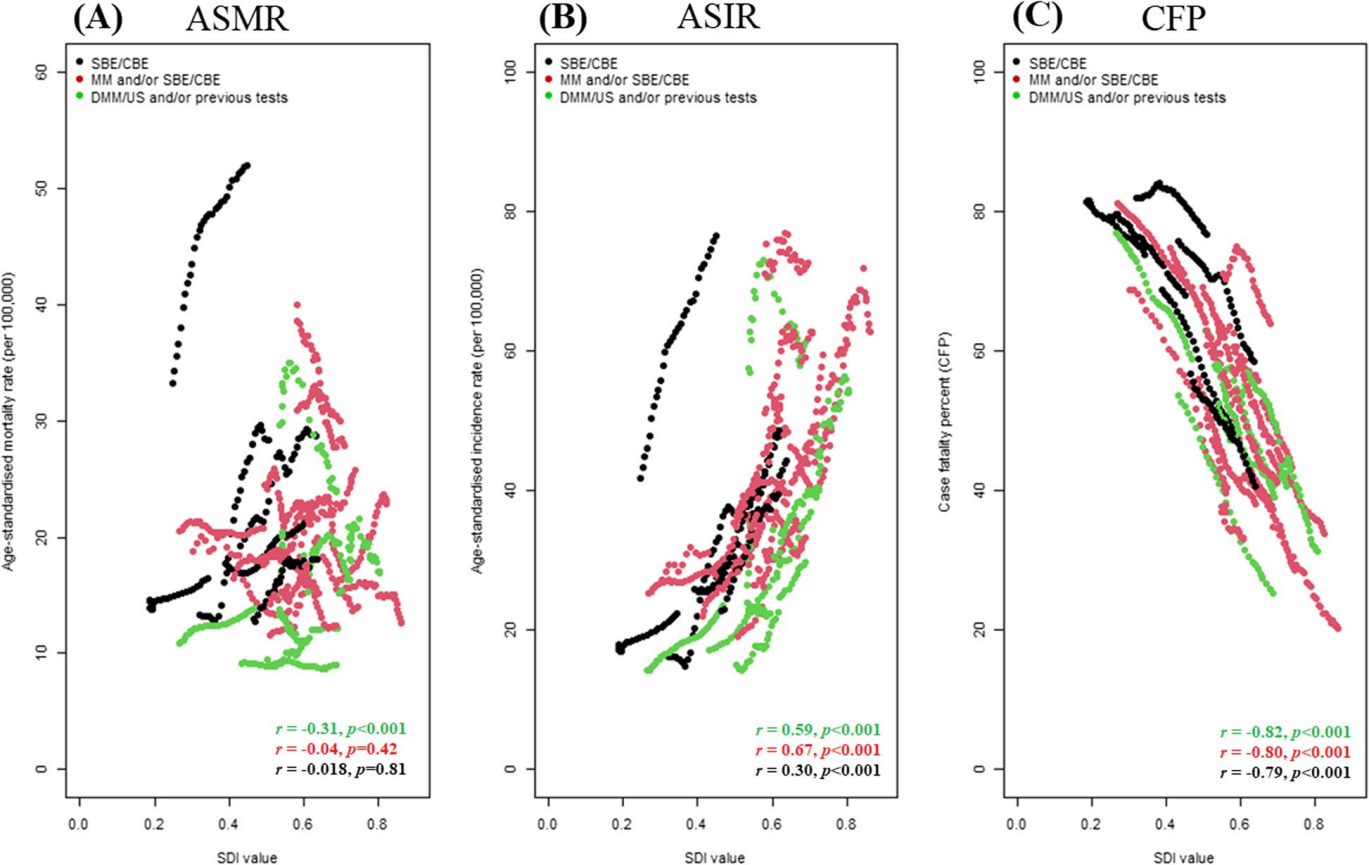
Relationship between breast cancer (BC) mortality, incidence, and case fatality (per 100,000 person-years) and countries’ sociodemographic index (SDI) by type of screening test among BRICS-plus, from 1990 to 2019. **A** For age-standardized mortality rate (ASMR). **B** For age-standardized incidence rate (ASIR). **C** For case fatality percent (CFP). CFP indicate age-standardized mortality to incidence ratio and multiply by 100. CBE, clinical breast examination; DMM, digital mammography; DMM/US, digital mammography and/or ultrasound; MM, mammography; SBE, self-breast examination; SBE/CBE tests, self-breast examination and/or clinical breast examination; US, ultrasound

Furthermore, based on each country’s sociodemographic index from 1990 to 2019, we found a strong negative link between CFP and SDI for nations using DMM/US and/or previous testing, while it has been discovered that incidence rates and SDI values are significantly positively correlated in nations having mammography and/or SBE/CBE (MM and/or SBE/CBE) programs (Fig. 6).

### Association of screening programs with BC outcome by BRICS-plus country-income levels

In order to investigate different patterns among BC screening programs, the data mentioned above was further divided by the income level/group of BRICS-plus countries. Notably, high-income and middle-income areas (in comparison to low-income areas) experienced a considerable difference in age-standardized BC mortality, DALYs, and case fatality rates when national BC screening programs were implemented, as opposed to having none or only pilot/opportunistic programs. Table 2 presents the relationship between breast cancer screening programs and age-standardized BC mortality, disability, and case fatality rates, segmented by country income levels.

**Table 2:** Mixed-effect multilevel regression to assess the relationship between age-standardized breast cancer mortality, disability, case fatality, and breast cancer screening (BCS) programs, by country-income levels (high-, middle-, low-income) across BRICS-plus

	**HICs**		**MICs**		**LICs**	
	**Estimate**	**95% CI**	**Estimate**	**95% CI**	**Estimate**	**95% CI**
**Mortality**						
No country/pilot screening program^a^	Reference		Reference		Reference	
National-wise screening program	− 13.10	− 16.21 to − 12.34	− 9.56	− 11.01 to − 8.99	2.11	− 2.03 to 9.65
Regional-wise screening program	− 10.41	− 15.32 to − 8.01	− 8.22	− 10.33 to − 7.56	− 1.43	− 2.44 to 8.54
**DALYs**						
No country/pilot screening program^a^	Reference		Reference		Reference	
National-wise screening program	− 196.82	− 202.65 to − 191.10	− 165.4	− 169.02 to − 163.76	137.77	− 87.41 to 159.20
Regional-wise screening program	− 123.41	− 126.61 to 121.23	− 112.87	− 114.45 to 86.77	108.44	− 99.23 to 111.46
**Case fatality (CF)**						
No country/pilot screening program^a^	Reference					
National-wise screening program	− 0.72	− 0.87 to − 0.63	− 0.61	− 0.69 to − 0.60	− 0.41	− 0.51 to 2.76
Regional-wise screening program	− 0.56	− 0.73 to − 0.41	− 0.43	− 0.44 to − 0.41	− 0.47	− 0.66 to 3.41

Models are equally adjusted as previous tables. 95 CI, 95% confidence interval; *DALYs*, disability-adjusted life years; *HICs*, high-income countries; *MICs*, middle-income countries; *LICs*, low-income countries

^a^No country program or existence of an opportunistic or pilot screening program

## Discussion

In addition to highlighting the association of risk factors and morbidities like high body mass index, smoking, second-hand smoke, diabetes, and CVD presence with the shape of mortality and DALYs in BC, our study shows that the availability of mammography (digital or traditional) and BCS are associated with breast cancer mortality, CFP, and DALYs in BRIC-plus countries. Age-standardized BC mortality and DALYs are lower in regions with well-established national and regional BCS initiatives. There are differences in mortality and morbidity among countries with different levels of economic income. The higher the SDI, the higher the standardized incidence of BC, and the lower the CFP after BC screening. Furthermore, this study extends our knowledge related to the influence of BCS types on case fatality and DALYs across BRICS-plus.

We further confirm that BCS is associated to reduce BC mortality and CFP. Consistent with previous studies, BC mortality is associated with high BMI, smoking, second-hand smoke, cardiovascular disease, and diabetes [49, 50]. Although the results do not anticipate an association between low physical activity and BC mortality, previous research has shown that regular physical activity also reduces the risk of death from BC [51], this might be due to variable confounding factors as every individual is different from other encompassing varied genetic, environmental, and health-linked risk factors. Modifiable risk factors for BC mainly include high BMI, smoking, alcohol consumption, low physical activity (PA), high fasting blood glucose, and a high-energy diet [52]. Given that the risk of BC-linked death is strongly associated with lifestyle factors, preventive measures can be taken to improve health considering reduced smoking and control diabetes and cholesterol levels. In addition to this community-based media or health education campaign link, “pink ribbon” can be conducted to educate people about the disease its consequences and ways to improve lifestyles in order to have better health outcomes. Large-scale studies come up concluding that obesity is a key player in postmenopausal breast cancer, reporting 6% increased risk for every 5 kg of body weight gain [53]. Therefore, with the advent of better screening techniques, timely preventive measures and improved BC treatment options BC mortality can be reduced.

We observed that BRICS-plus nations in 2019 that used digital screening tests and/or previous tests (DMM/US and/or previous tests) were most of middle-income countries (MICs) as well as high-income countries (HICs) and had lower age-standardized DALYs than other screening programs. As previously reported, we also found significant effect of smoking and high BMI [54] on age-adjusted DALYs; particularly low physical activity (PA) was associated with lower breast cancer DALYs. National or regional BCS programs also affected age-standardized BC disability. One of the studies from China showed that if BCS coverage remained the same (25.7%), breast cancer DALYs in women were projected to increase by 26.7%, which may be related to risk factors such as aging, high body mass index, smoking, and environment. In addition, the effect of BCS on mortality risk may be insufficient in a short period of time. However, with the strong support of the government, the scope of BCS in China is still gradually expanding [55]. Given BRICS-plus’s population size, widespread coverage is unlikely to be achieved in the near future. Therefore, compared with opportunistic screening, secondary prevention strategies such as population-based screening should be actively promoted.

It is worth noting that only mastering the correct breast self-examination method can help the clinical detection rate. Therefore, clinical breast examination CBE remains an important tool for early detection, diagnosis, and surveillance, especially in subgroups of women at high risk of breast cancer [2]. Therefore, two or more methods can be used for BCS to improve the efficiency of screening results and ensure early detection and intervention of breast cancer.

From the perspective of different SDI regions in the world, the higher the SDI, the higher the standardized incidence rate, and the higher the SDI, the lower the CFP, which is consistent with the research results of different income levels in the BRICS-plus countries [56]. Our study also highlights the importance of national and regional screening programs which can reduce BC mortality compared with no country/pilot screening programs. However, this finding was not reflected in LICs, which may also be related to the lack of local data sources. Population-based mammography screening programs shown to reduce breast cancer mortality [57]. India, China, and Russia report large differences in breast cancer survival between regions, indicating inequities in access to diagnostic and treatment services in these vast countries. BRICS-plus could consider changing breast screening guidelines. If it is not possible in economically underdeveloped areas, breast self-examination can be recommended to improve women's awareness of breast health care, and if breast lumps are found, visit the clinic in time. Primary care physicians should be trained to perform high-quality clinical examinations of symptomatic women. In countries with poorer economic conditions where CBE training is being implemented, systematic CBE with appropriate training has a high negative predictive value [58]. BRICS-plus need to recognize the importance of quality-assured population screening, which is a hard task in these densely populated countries.

## Limitation

Here, we like to mention some of the limitations in our study. First, the different data sources and collection methods in GBD will inevitably affect the quality of data and the reliability of results. Some of this data may be inaccurate, as we do not know whether the BCS plans presented on paper are actually being implemented and what the BCS adoption rate is in each country. Secondly, current ecological study design comprehends at population level, so ecological bias should be considered. Third, there is a lack of data on breast cancer subtypes in GBD, so the association of BCS with its subtypes could not be addressed. Fourth, there are different treatment strategies including surgery and drug treatments for breast cancer. Impact on mortality can vary and such information was unknown and should be added in the future. Despite the limitations of the research, the GBD database is still one of the few databases that can provide a global comparison of breast cancer burden and able to provide effective recommendations for BC prevention and control in BRICS-plus.

## Conclusions

The gradual privatization of health care has led to rising inequality, fragmentation of public health services, and high levels of public spending, which are common characteristics of the BRICS countries. The recent BRICS-plus analysis adds to the claim that BCS has good effects on age-standardized mortality, DALY rates, and case fatality percentage for female breast cancer. Therefore, it is mandatory to arrange mammography screening for BCS at the national level and introduce interventions for BCS-related risk factors to effectively reduce risk factors and comorbidities associated with BC mortality and DALYs. In addition, as emphasized in the BRIC-plus BCS and diagnostic guidelines, BCS must optimize benefits, reduce mortality, and balance false-positive and false-negative rates. Therefore, our analysis helps policymakers to focus on establishing goals within organized BCS initiatives in order to reduce BC mortality and disability. To save the lives of the thousands of women currently dying from treatable malignancies, policymakers must be more decisive and rational in their investments.

### Supplementary Information

**Additional file 1:** **Table S1.** Breast cancer screening program information for BRICS-plus countries.

## Data Availability

The dataset analyzed during the current study are available in the Institute for Health Metrics and Evaluation (IHME): http://ghdx.healthdata.org/gbd-results-tool.

## Abbreviations

BC: Breast cancer
BCS: Breast cancer screening
GBD: Global Burden of Disease
SDI: Sociodemographic index
DR: Death rates
DALYs: Disability-adjusted life years
CVD: Cardiovascular disease
BRICS: Brazil, Russia, India, China, South Africa
BMI: Body mass index
SBE: Self breast examination
CBE: Clinical breast examination
US: Ultrasound
SBI: Society of Breast Imaging
ACR: American Cancer Research
YLL: Year of life lost
YLD: Year lived with disability
CFP: Case fatality percent
PA: Physical activity
