# Supplementary material for: Estimating disparities in breast cancer screening programs towards mortality, case fatality, and DALYs across BRICS-plus
Source: BMC Med. 2023 Sep 1;21:299. doi: 10.1186/s12916-023-03004-4 (PMC10472654; doi:10.1186/s12916-023-03004-4)
Supplement: Supplementary file 1 — Additional file 1: Table S1. Breast cancer screening program information for BRICS-plus countries. [file 12916_2023_3004_MOESM1_ESM.pdf]

Estimating disparities in breast cancer screening programs towards mortality, case fatality and DALYs across BRICS-Plus

Supplementary Table 1. Breast cancer screening programe information for BRICS-Plus countries

sn	countries	national/regional	start of programe	national coverage	Examination used	age range	
1	Afghanistan	National	2017	-	SBE	-	
2	Armenia	National	2015	-	MM, US	-	
3	Bangladesh	National	2017	-	-	-	
4	Bhutan	National	2015	-	-	-	
5	Brunei	National	2019	-	MM	40-69	
6	Cambodia	Regional	2017	-	MM, US		
7	China	Regional	2009	-	CBE, US	35-59	
			2012	-	SBE, CBE, US	35-64	
8	India	National	2015	-	-	-	
9	Indonesia	National	2015	-	-	-	
10	Kazakhstan	National	2008	2013	DM	50-60	
11	Kyrgyzstan	National	2007	2007	DM	40-69	
12	Laos	Regional	2014	-	CBE, MM	-	
13	Malaysia	Regional	2011	-	CBE, MM	40-74	
14	Maldives	National	2019	-	CBE	20+	
					MM	40+	
15	Myanmar	No	-	-	-	-	
16	Nepal	No	-	-	-	-	
17	Pakistan	Regional	2016	-	SBE, CBE	40+	
18	Philippines	Regional	2011	-	MM	25+	
					SBE	50+	

19	Singapore	National	2002	-	BSE, +/-MM	40+	
					BSE, MM	50+	
20	Sri Lanka	National	2015	-	CBE, MM, US	-	
21	Thailand	National	2019	-	MM	30+	
22	Vietnam	Regional	2008	-	SBE, CBE	40-55	
23	Botswana	Regional	2011	-	SBE, CBE	-	MM for high income people
24	Eswatini	Started policy making	2019	-	-	-	
25	Lesotho	Regional	2013		CBE	25+	
26	Namibia	Regional	2013		-	-	Educated or insured people
27	South Africa	National	2013	-	BSE, CBE	All women	
					MM	40+	
28	Belarus	National	1991	2014	CBE, MM	50-69	
29	Russia	Regional	2007-2012	-	DM	>40	
30	Argentina	Regional	2005	-	CBE	40-50	
					MM	50-70	
31	Bolivia	Regional	2013	-	SBE, CBE	25-64	
					MM - private only		
32	Brazil	Regional	2008	-	CBE, MM	50-69	
33	Paraguay	Opportunistic Program	2013	-	CBE	40-69	
34	Uruguay	Regional	1996 (pilot)		BSE, CBE, MM	45+	
35	Venezuela	Regional NGO	2013	-	BSE, CBE, MM	35-74	
